# Acute aortic wall injury caused by aortic cross-clamping: morphological and biomechanical study of the aorta in a swine model of three aortic surgery approaches

**DOI:** 10.1590/1677-5449.190025

**Published:** 2020-03-12

**Authors:** Marcela Polachini Prata, Rodrigo Gibin Jaldin, Pedro Luiz Toledo de Arruda Lourenção, Marcone Lima Sobreira, Ricardo de Alvarenga Yoshida, Simone Antunes Terra, Rosa Marlene Viero, Winston Bonetti Yoshida

**Affiliations:** 1 Universidade Estadual Paulista – UNESP, Faculdade de Medicina de Botucatu, São Paulo, SP, Brasil.

**Keywords:** abdominal aorta, tensile strength, mechanical stress, vascular closure devices

## Abstract

**Background:**

Aortic cross-clamping and balloon occlusion of the aorta could lead to damage to the aorta wall.

**Objective:**

The aim of this study was to investigate changes to the aorta wall related to the method used to interrupt flow (clamping or balloon) in the different techniques available for aortic surgery.

**Methods:**

Experiments were performed on 40 female pigs, weighing 25-30kg, which were randomly allocated to 4 study groups: S (n=10), no intervention (sham group); C (n=10), midline transperitoneal laparotomy for infrarenal abdominal aortic access with 60 min of cross-clamping; L (n=10), laparoscopic infrarenal abdominal aortic surgery with 60 min of cross-clamping; EV (n=10), remote proximal aortic control with transfemoral arterial insertion of aortic occlusion balloon catheter, inflated to provide continued aortic occlusion for 60min. After euthanasia, the aortas were removed and cross-sectioned to obtain histological specimens for light microscopic and morphometric analyses. The remaining longitudinal segments were stretched to rupture and mechanical parameters were determined.

**Results:**

We observed a reduction in the yield point of the abdominal aorta, decrease in stiffness and in failure load in the aortic cross-clamping groups (C and L) compared with the EV group.

**Conclusions:**

Aortic cross-clamping during open or laparoscopic surgery can affect the mechanical properties of the aorta leading to decrease in resistance of the aorta wall, without structural changes in aorta wall histology.

## INTRODUCTION

Choosing the most appropriate surgical approach for repair of an infrarenal abdominal aortic aneurysm (AAA) involves analysis of surgical risk, comorbidities, morphology of the AAA, patient life expectancy, experience of the surgical team with each technique, and the scientific evidence for each technique. Strategies currently available for treatment of this condition disease include conventional open surgery, videolaparoscopic surgery, and endovascular treatment.^1^^-^ 4 Despite the initial enthusiasm for laparoscopic aortic surgery, the technique has not been widely adopted in vascular surgery because of the challenges inherent to the procedure and the long learning curve, so its use has remained restricted to a few specialized centers.^2^^,^ 3 There is evidence to suggest that elective laparoscopic surgery to repair an AAA has comparable invasivity to endovascular repair (EVAR), with the advantages of a lower conversion rate and similar morbidity and mortality, while offering a minimally invasive option for treatment of patients with anatomy that is unsuitable for EVAR.^1^^,^ 3 ^,^ 4

Conventional and laparoscopic surgery both involve use of hemostatic clamps (atraumatic clamps) to control blood flow and reflux. However, despite their “atraumatic” label, these clamps cause acute injury to the artery wall. The degree of injury appears to be dependent on the pressure applied and the duration of clamping, and ranges from distortion of the intima to complete breakdown of the tunica media of the vessel, with weakening of the artery wall, intimal hyperplasia, and restenosis.^5^ Margovsky et al.^6^ observed formation of cavities in the tunica media, a change known as cystic necrosis of the media that is found in degenerative processes involving the aorta, such as aortic dissections, degenerative aneurysms, and aging.^7^^,^ 8 Loh et al.^9^ described acute rupture of the abdominal aorta provoked by clamping, documenting localized ischemic parietal injury that weakened the structural integrity of the aorta. Nevertheless, it has been observed that, although clamping caused morphological changes,^10^ there were no significant changes to the mechanical properties of the artery wall over the long term.^11^ However, there is a lack of studies that correlate the acute changes caused by clamping of the aorta with mechanical changes to its walls.

While the aorta is not clamped during routine endovascular treatment, there is temporary occlusion of the aortic flow by devices for deployment of stent-grafts and by inflation of the balloon used for positioning the device after release.^12^^,^ 13 The negative effects on the mechanical properties of the aorta wall of oversizing stent-grafts has already been studied.^14^ There are also clinical scenarios in which prolonged balloon inflation is needed in the absence of the effects of contact between an endoprosthesis and the artery wall – for example in resuscitative endovascular balloon occlusion of the aorta (REBOA), used for treatment of a ruptured aorta or for intraoperative complications.^13^^,^ 15 Injuries caused by intraluminal inflation of the balloon range from endothelial damage (abrasion and dissection) to necrosis of the tunica media, chiefly interfering in the function of components of the extracellular matrix.^16^ Keris et al.^17^ observed that arterial segments subjected to balloon angioplasty had reduced tangential elastic modulus in the circumferential direction, which could predispose to increases in the diameter of the vessel when subjected to normal blood pressure.

Although it appears that less invasive methods are beneficial in terms of the systemic repercussions of conventional surgical trauma, studies are needed that can shed light on the body’s pathophysiologic responses after open surgery, endovascular repair, or videolaparoscopic surgery on the aorta.^3^ The objective of this study was to conduct comparative assessments of the structural and biomechanical changes to the aorta wall provoked by methods used to temporarily interrupt flow through the aorta, depending on the surgical access used to approach the aorta.

## MATERIALS AND METHODS

A prospective, randomized, experimental study was conducted. The study complies with the Guide for Care and Use of Laboratory Animals and was approved by the institution’s Animal Experimentation Ethics Committee (protocol 899-2011). Female pigs, Large White - Landrace cross, weighing 25 to 30 kg were used. After an adaptation period of 5 to 10 days, the animals were allocated at random by simple lots to one of three experimental groups, with 10 animals in each: Group C (open surgery), Group L (videolaparoscopy), or Group EV (endovascular surgery). An additional group, Group S (Sham), was made up of aorta specimens from 10 animals with the same origin and weight range that were removed by the study soon after slaughter at the abattoir used by the farm that reared them. This group was used as the standard of normality for biomechanical and histological parameters.

### Anesthesia procedures

The animals were kept in preoperative fasting for 8 hours. Premedication comprised a combination of 0.1 mg/kg of acepromazine 1%, 8 mg/kg of ketamine, 0.5 mg/kg of xylazine, and 0.5 mg/kg of morphine, via intramuscular injection. Fifteen minutes after premedication, the central vein of the ear was cannulated and used for anesthesia induction by administration of 2 mg/kg of ketamine and 2 mg/kg of diazepam. The animal was then placed in the prone position on the operating table for oral endotracheal intubation. Anesthesia was maintained with isoflurane at 5-10 mL/kg/min. Mechanical ventilation was provided with a tidal volume of 12-15 mL/kg of oxygen, at a rate of 10 to 12 respiratory movements/min, to maintain expiratory carbon dioxide pressure in the range of 35 to 45 mmHg. Baseline hydration was maintained with Ringer’s lactate solution infused at 5 mL/kg/h with an intravenous infusion pump and complemented with infusion of saline 0.9% according to hemodynamic requirements identified by a veterinary anesthetist throughout the procedure. Intraoperative monitoring comprised pulse oximetry with a sensor placed on the animal’s tongue, a rectal thermometer for body temperature, and invasive blood pressure monitoring via a carotid access, with arterial catheterization using an 11 cm 6F introducer.

### Surgical procedures

**Group C:** Animals were positioned on the operating table in horizontal dorsal decubitus. After antisepsis and draping of the surgical field, a midline laparotomy was performed with transperitoneal exposure of the aorta. The infrarenal aorta was exposed from the point it crosses the left renal vein and the origin of the renal arteries and the aortic bifurcation were identified. The infrarenal aorta was then clamped with Debakey atraumatic forceps to interrupt flow through the aorta for 60 minutes.

**Figure 1:**
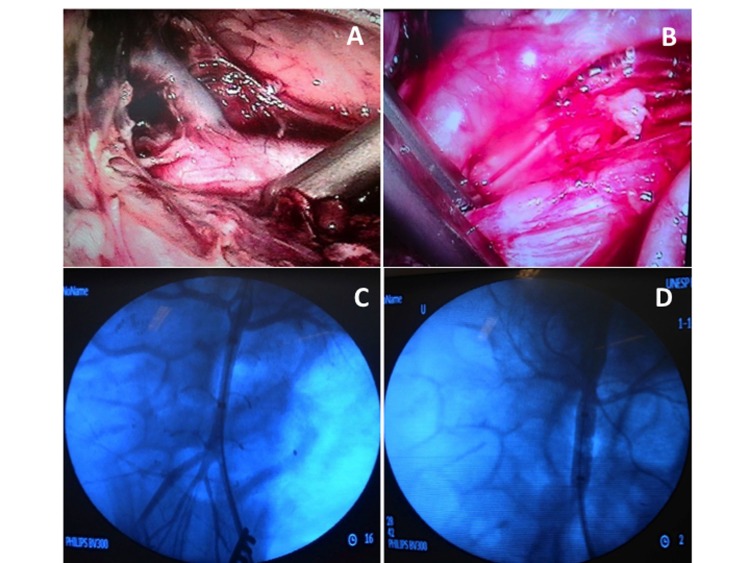
Site of interruption of flow through the aorta for minimally invasive techniques. (A) Laparoscopic dissection and identification of the left renal artery; (B) Placement of the laparoscopic hemostatic forceps; (C) Aortography to identify emergence of the renal arteries; (D) Aortography with the balloon inflated.

**Group L:** Animals were placed on the operating table in right lateral decubitus. Antiseptic solution was applied and the surgical field was draped. The pneumoperitoneum procedure was initiated via a percutaneous puncture with a Veress needle. After pneumoperitoneum was established with CO_2_ at a pressure of 16 mmHg, an 11 mm trocar was positioned lateral to the umbilical scar to introduce a 30° optical lens. After reestablishing pressure at 12 mmHg, two further 11 mm trocars were positioned lateral to the midline, above and below the line of the umbilicus. Another three 11 mm trocars were placed along the left side of the abdominal wall, using the costal margin, the midaxillary line, the large dorsal muscle, and the iliac crest as references. Exposure of the aorta began by medial elongation of the left colon, the left kidney, and the splenic flexure, using laparoscopic graspers, scissors, and harmonic scalpel (Ultracision®, Johnson & Johnson®). After completing dissection of the abdominal aorta, a laparoscopic aortic clamp (Storz®) was applied immediately below the left renal artery to interrupt flow through the aorta for 60 minutes (Figure 1).

**Group EV:** Animals were positioned on the operating table in horizontal dorsal decubitus. After antisepsis of the groin, the surgical field was draped. A transverse inguinotomy was performed and the left common femoral artery was accessed. Under direct view, the common femoral artery was punctured with a 21G single-wall needle and a rigid metal “J” tip guidewire inserted, enabling arterial catheterization with an 11 cm 6F introducer using the Seldinger technique. A 260 cm 0.035” Roadrunner hydrophilic guidewire (Cook Medical®) was advanced to the infrarenal aorta under radiographic guidance (Phillips®, BV 300 C-arm, United States), then a 5F Pig Tail angiographic catheter (Cook Medical®) was inserted to conduct aortography with injection of 20 mL of Optiray® nonionic contrast, delineating the renal arteries by road mapping. Aortography was performed before and after inflation of the balloon, with a total contrast volume of 40 mL. After removal of the introducer and provoking hemostasis by manual compression, the angiographic catheter was substituted for a complacent balloon catheter with a diameter of 32 mm for aortic occlusion (Coda Balloon Catheter®, Cook Medical®, USA). The angiographic catheter was then inserted via the carotid access. After positioning the balloon just below the origin of the renal arteries, it was inflated until flow was entirely interrupted, using 15 mL of contrast solution, with angiographic control, and maintained inflated for 60 minutes (Figure 1).

### Experimental protocol

All of the surgical procedures were conducted by the same team, following the same experimental sequence. Prior to aortic clamping, sodium heparin was administrated intravenously at a dosage of 100 UI/kg to animals in all groups. In all groups, the duration of interruption of aortic blood flow was 60 minutes. After this period, animals were euthanized by anesthetic overdose and median laparotomy was performed to access the aorta and remove specimens for study. Segments of abdominal aorta approximately 5 mm in length were taken from the site of clamping/ballooning from five animals per group, including the point at which the clamp/balloon had been positioned, and fixed in buffered formalin 10% for histological study at a later date. From the other five animals in each group, samples of the aorta measuring approximately 3 cm (1 suprarenal cm and 2 infrarenal cm, to include the point where flow had been interrupted) were taken for use in the tensile strength tests.

### Histology

Segments of aorta preserved in formol were processed in a Leica TP102 tissue processor and set in paraffin blocks in a Leica EG 1160. Posteriorly, serial cross-sections of approximately 5 µm were cut in a Leica RM 2155 microtome, mounted on glass slides and stained with hematoxylin and eosin (H & E) and Picrosirius Red, for collagen, and Verhoeff’s stain, for analysis of elastic fibers in the aorta wall. General changes to the vascular wall were recorded, such as loss of the lamellar architecture, reduction of smooth muscle cells, mononuclear cellular infiltrate, disorganization of collagen fibers, intensity of Picrosirius staining, and reduction or fragmentation of elastic fibers.

### Biomechanical tests

**Figure 2:**
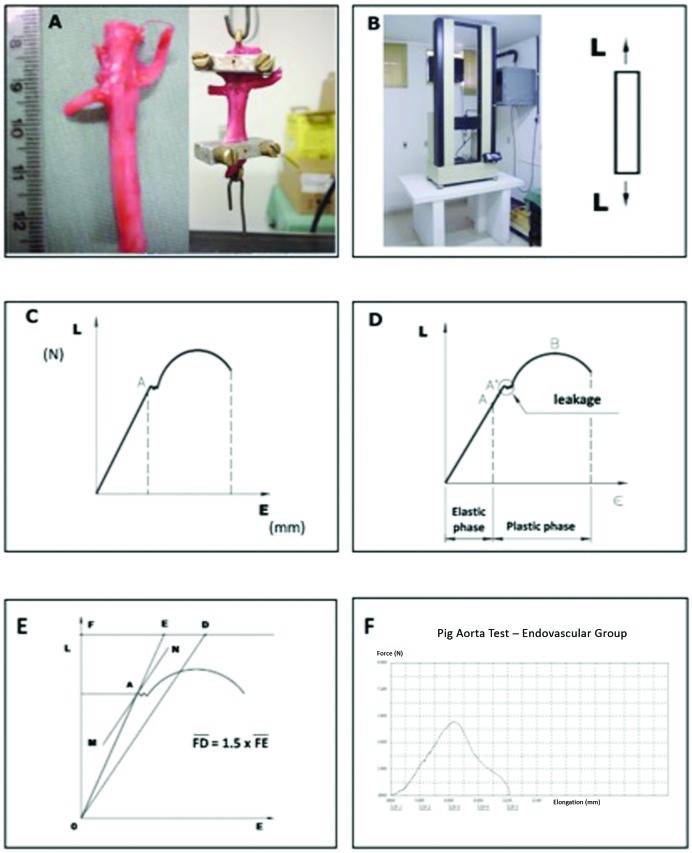
Mathematical model used for tensile testing of the aorta. (A) Segment of aorta including the clamped portion, fixed in the machine’s jaws; (B) Universal Mechanical Test Machine in the Biomechanical Testing Laboratory at the Surgical Techniques and Experimental Surgery Laboratory and the uniaxial force vector applied to the sample; (C) Load vs. elongation diagram; (D) Elastic and plastic phases on load vs. elongation diagram; (E) Elastic limit calculated by the Johnson method; (F) Example of a graph from the test.

The segments of abdominal aorta, including the portions immediately above and immediately below the point at which flow had been interrupted, were subjected to destructive uniaxial tensile testing, using a method employed previously^8^^,^ 18 ^-^ 20 for evaluation of mechanical properties. The ends of each segment were fixed using the machine’s clamps, which are smooth, non-cutting, metal plates, enabling the aortic segment to be stretched longitudinally. The traction velocity adopted was 30 mm/min. The apparatus employed was an EMIC® Universal Mechanical Test Machine, model DL 10.000 (Equipments and Testing Systems, Ltd., Curitiba, PR, Brazil), which is a system with precision of ± 0.018+F/3700 KN, as tested according to the Brazilian Association for Technical Standards (ABNT) NBR6156 and NBR6674 specifications. The machine operates in conjunction with a microcomputer with the Windows 98® operating system installed, running Mtest 1.00 software. At the end of the test, the program provides values for the mechanical properties chosen by the user and a load vs. elongation graph. These diagrams can be used to derive the following parameters (Figure 2): **Yield point (N)**: maximum load value at which the material still has the capacity to return to its original length if the load is removed; graphically, this corresponds to the maximum tension value at which the linear function of the load-elongation curve still obeys Hooke’s Law, calculated using the Johnson method; **Coefficient of stiffness (N/mm)**: force (N) divided by elongation (mm) at the elastic limit; which, since it is a constant, linear, numeric relationship, represents a material’s deformation capacity as the load is applied; **Maximum load – force at failure (N):** the greatest load withstood by the material before rupture, i.e., the limit of resistance.

### Statistical analysis

The sample size of 10 animals per experimental group was calculated with the help of the institution’s Research Support Office on the basis of previous experimental studies of aortic surgery using porcine models,^21^^-^ 24 and was adopted as the reference for constituting the groups. First, normality of the data was tested, showing that they were symmetrical. Therefore, analysis of variance (ANOVA), followed by the Tukey test was used for multiple comparisons to test whether there were differences between the C, L, EV, and S groups.

## RESULTS

### Biomechanical tests

**Figure 3:**
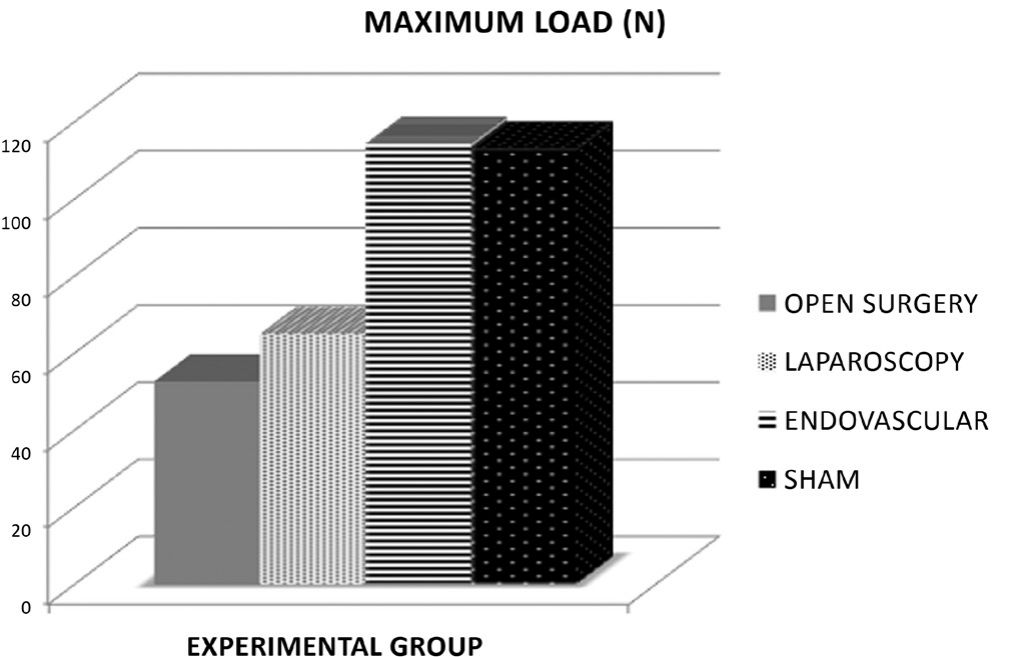
Absolute comparison between groups for the parameter maximum load, where the difference in maximum load was statistically significant (p < 0.05) between groups EV and L and between EV and C.

**Figure 4:**
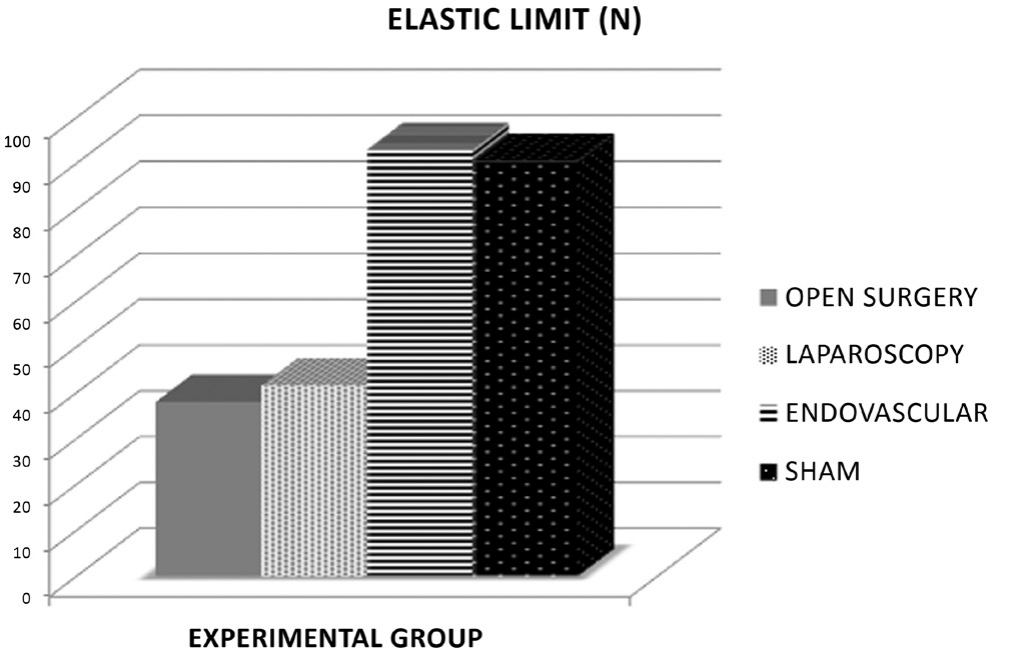
Absolute comparison between groups for the parameter elasticity, where the difference in elastic limit was statistically significant (p < 0.05) between groups EV and L and between EV and C.

**Figure 5:**
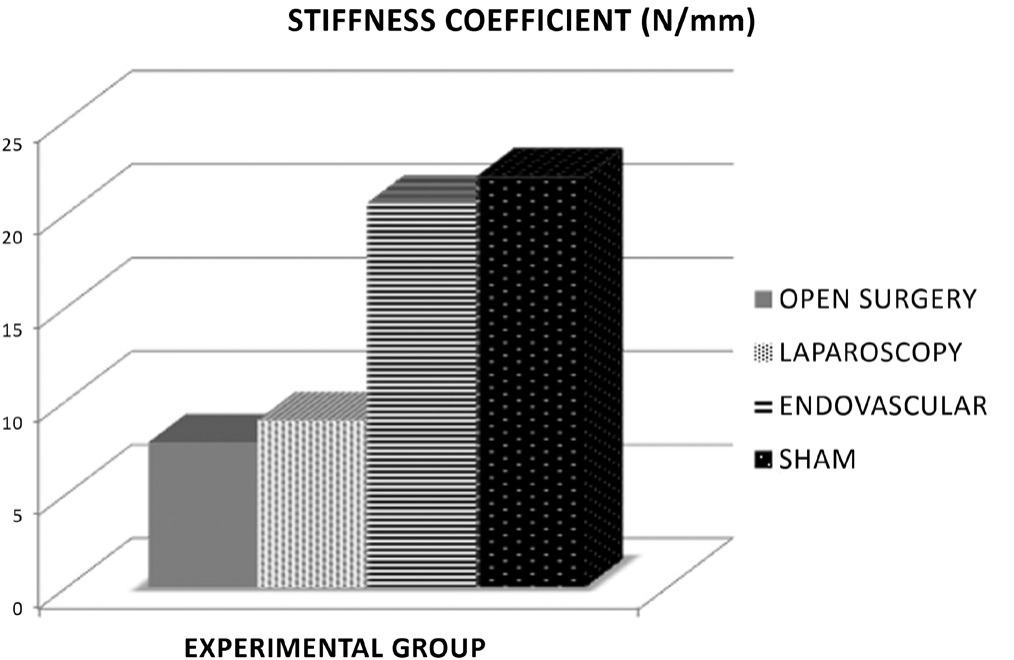
Absolute comparison between groups for the parameter stiffness coefficient, where the difference in stiffness coefficient was statistically significant (p < 0.05) between groups EV and L and between EV and C.

The EV group exhibited the greatest resistance to load, with higher stiffness coefficient (p < 0.05), maximum load (p < 0.05), and yield point (p < 0.05), than groups C and L. The aorta samples from the EV group had similar mechanical behavior to the S group in the tensile tests (Figures 3, 4, and 5).

### Histological analysis of the aorta specimens

**Figure 6:**
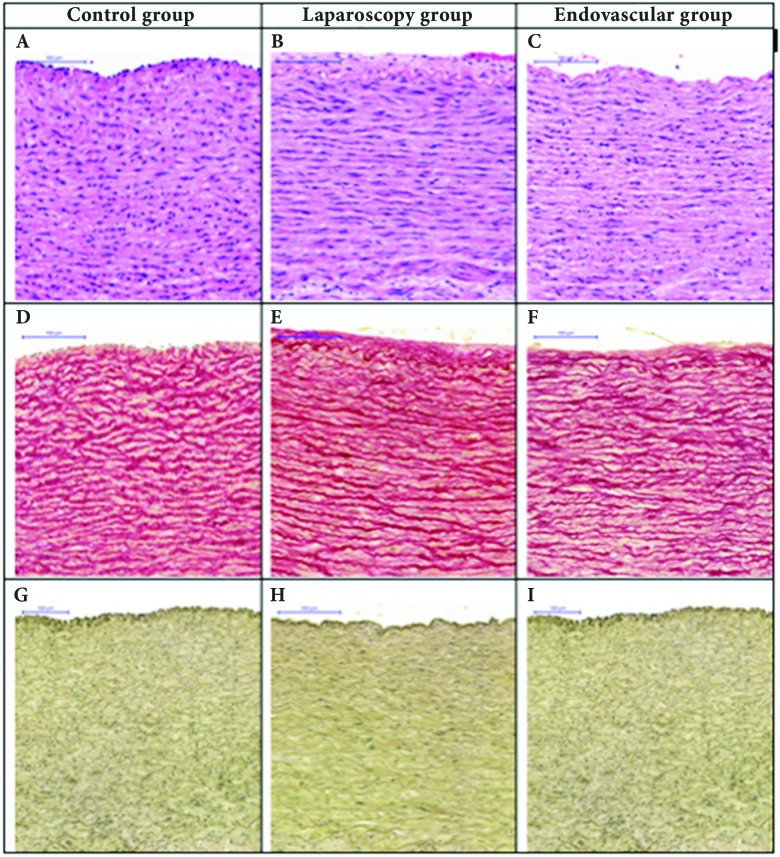
Histological sections of abdominal aorta. (A, B, C). The lamellar arrangement is preserved, characterized by the parallel pattern of the fibers of the tunica media. Number and nuclei of smooth muscle cells are preserved. Absence of mononuclear inflammatory infiltrate (hematoxylin and eosin, 200x); (D, E, F) Collagen fibers with normal organization and staining (Picrosirius, 200x); (G, H, I) Sinuous elastic fibers present throughout the vascular segment, without fragmentation and in normal quantities (Verhoeff, 200x).

The histology of the aorta specimens was preserved in all of the cases analyzed. No changes were observed in cellular structure, collagen fibers, or elastic fibers in the samples assessed, irrespective of study group (Figure 6).

## DISCUSSION

The main findings of this study consist of identification of changes provoked by the hemostatic surgical forceps at the clamping site, which is unavoidable during aortic surgery. Whenever a vessel is manipulated, there is a possibility of plaque rupture, intimal injury, and formation of thrombi during and after placement of the hemostatic clamps. Even after endovascular surgery, 1 hour after arterial ballooning for angioplasty, artery wall changes have already occurred, such as: endothelial denudation, deposition of platelets, mural thrombi, and endothelial tears involving the internal elastic lamina.^25^ The balloon used during EVAR is complacent, but it is kept inflated above the animal’s blood pressure for a long time, and could therefore be a source of artery wall injury. When it undergoes balloon angioplasty, it is subjected to radial tensions that exceed its physiological range and so damage could occur, in particular to collagen fibers.^26^ The balloon’s complacency causes the area of contact between the balloon and the aorta wall to be greater than its nominal surface, because it accommodates to the smaller diameter of the pig aorta. Experiments have demonstrated that this injury will induce thickening of the wall of the vessel and will be determined by the stress at the surface of the vessel wall that is in contact with the balloon.^27^ Consigny et al.^28^ observed an immediate increase in arterial diameter, endothelial denudation, injuries to smooth muscle cells, reduced arterial thickness, and increased elastic modulus soon after arterial ballooning. In the present study, the aortic segment in contact with the balloon could have been a point of localized ischemic changes, caused by compression of the vasa vasorum, by a lack of contact between the aorta and circulating blood, and by reperfusion.

The hypothesis of acute changes to the aorta wall after prolonged ballooning is not without foundation, even using a complacent balloon, since it is inflated at high pressure and for a long time. However, the mechanical behavior of the aortas in which flow was interrupted by balloon inflation was similar to those from the normal controls. These results may suggest that inflation of the complacent balloon inside the aorta, even for a prolonged period, does not provoke structural changes in the wall, which is fundamental to the durability of endovascular techniques. Increased aortic diameter due to a possible injury and weakening of the wall at the site of ballooning (the proximal anchorage for endoprostheses) could lead to degeneration of the neck and consequent type I endoleaks and stent migration.^29^ Additionally, endovascular hemostasis is increasingly achieved during aortic emergencies using an intraluminal balloon and the REBOA technique,^30^ so it is important to accumulate data showing that this technique does not provoke persistent mechanical changes to the aorta wall.

Analyses showed that the mechanical parameters of specimens from groups C and L were inferior to those of specimens from the EV group, whereas the biomechanical variables for the S group were similar to those observed for the EV group, revealing a reduction in the resistance of the aorta wall after use of hemostatic clamps. The distensibility of the aorta is dependent on components in the tunica media – collagen, elastin, and proteoglycanes – and all of these can affect its resistance to traction,^8^ since the elastic capacity of cardiovascular tissues is directly related to their biomechanical behavior.^18^ In the final analysis, tests of tensile resistance reflect the stiffness and the elasticity of the aorta, i.e., they analyze the capacity of collagen and elastin to enable the aorta to distend, which is a fundamental element in its function.^8^ Maximum load, yield point, and stiffness coefficient are the parameters most related to these biomechanical properties. It is possible that these factors contributed to the changes observed in the C and L groups during the biomechanical tests, since the mechanical tension in the artery wall is dependent on the load applied and the deformed vascular geometry.^31^^-^ 33

Since the biomechanical parameters of the artery wall are to a great extent due to its collagen and elastic fibers, stains specific to these components of the wall were included, but even so, no marked and significant changes were observed in these slides under light microscopy. Borges et al.^7^ demonstrated that Picrosirius Red analyzed under polarized light, together with conventional light microscopy would be the best method for evaluating the structure of collagen, since it allows the arrangement and grouping of collagen fibers to be studied, because of its normal birefringence. This stain can be used to view the morphology of intact collagen bundles and fragmented bundles and collagenolysis can also be detected. It is possible that using polarized light would have shown some type of rearrangement of the three-dimensional structure of the collagen fibers that was not detected with the conventional techniques employed.

The histological analyses were not sufficiently sensitive to detect acute structural changes in the components of the aorta wall, but functional or ultrastructural changes may have occurred. Studies with ultramicroscopy, with histochemistry for other components of the media^25^ and with immunohistochemistry for elastases could possibly have shown some type of change that would have confirmed the biomechanical changes observed. There are studies that suggest that ultrastructural damage to the artery wall is provoked by clamping after surgery, even in the absence of histological damage identifiable under light microscopy.^34^^,^ 35 Hemostatic forceps have grooves that exert significant local pressure on the artery wall, which invariably results in trauma to the vessel.^24^ The pressure exerted by the clamp on the aorta wall, in addition to local transitory ischemia followed by reperfusion, could have provoked changes to the vascular structure and biomechanical parameters. Although the acute changes to the aorta wall caused by clamping have been documented in many studies, apparently they do not result in permanent weakening of the vessel.^36^ Dobrin et al.^37^ described persistent injuries to the clamped area for up to 6 months afterwards, but they were not associated with chronic mechanical changes to the aorta. These findings could explain the safety of these techniques that have been used for decades.

## CONCLUSIONS

Use of clamping during open or laparoscopic surgery causes acute mechanical changes to the aorta suggestive of reduced resistance, even without apparent morphological changes. Prolonged inflation of the intraluminal balloon did not change the mechanical properties of the wall, denoting maintenance of its structural integrity.
