# Supplementary material for: Acute aortic wall injury caused by aortic cross-clamping: morphological and biomechanical study of the aorta in a swine model of three aortic surgery approaches
Source: J Vasc Bras. 2020 Mar 12;19:e20190025. doi: 10.1590/1677-5449.190025 (PMC7244208; doi:10.1590/1677-5449.190025)
Supplement: Translation

# Translation: Lesão aguda da parede arterial provocada pelo método de interrupção temporária de fluxo em diferentes vias de cirurgia aórtica: estudo morfológico e biomecânico da aorta de porcos

**Authors:** Marcela Polachini Prata, Rodrigo Gibin Jaldin, Pedro Luiz Toledo de Arruda Lourenção, Marcone Lima Sobreira, Ricardo de Alvarenga Yoshida, Simone Antunes Terra, Rosa Marlene Viero, Winston Bonetti Yoshida

**Affiliations:** 1 Universidade Estadual Paulista – UNESP, Faculdade de Medicina de Botucatu, São Paulo, SP, Brasil.

**Keywords:** aorta abdominal, resistência à tração, estresse mecânico, dispositivos de oclusão vascular

## Abstract

**Contexto:**

O clampeamento aórtico e a oclusão da aorta com balão poderiam levar a lesões na parede aórtica.

**Objetivo:**

O objetivo deste estudo foi verificar as alterações da parede aórtica relacionadas ao método de interrupção de fluxo (cample ou balão) em diferentes técnicas disponíveis para cirurgia de aorta.

**Métodos:**

Os experimentos foram realizados em 40 porcos fêmeas pesando de 25-30 kg, alocados para quatro grupos: S (n = 10), nenhuma intervenção (*sham)*; C (n = 10), laparotomia mediana transperitoneal para acesso à aorta abdominal infrarrenal com tempo de clampeamento de 60 minutos; L (n = 10), cirurgia laparoscópica da aorta abdominal infrarrenal com tempo de clampeamento de 60 minutos; EV (n = 10), controle aórtico proximal com inserção de cateter-balão para oclusão aórtica por acesso femoral, inflado a fim de promover oclusão aórtica contínua por 60 minutos. Após a eutanásia, as aortas foram removidas e seccionadas para obtenção de espécimes histológicos destinados a análises morfométricas e por microscopia de luz. Os fragmentos longitudinais restantes foram estirados até a ruptura, e determinaram-se padrões mecânicos.

**Resultados:**

Observou-se redução do limite de proporcionalidade da aorta abdominal, diminuição da rigidez e da carga de ruptura nos grupos submetidos a campleamento aórtico (C e L) em comparação ao grupo EV.

**Conclusões:**

O campleamento aórtico durante cirurgia aberta ou laparoscópica pode afetar as propriedades mecânicas da aorta, ocasionando redução de resistência da parede aórtica sem desencadear alterações na estrutura histológica da parede aórtica.

## INTRODUÇÃO

A escolha da via cirúrgica mais adequada para a correção do aneurisma de aorta abdominal (AAA) infrarrenal envolve análise do risco cirúrgico, comorbidades, morfologia do AAA, expectativa de vida do paciente, experiência da equipe cirúrgica com a técnica, e evidências científicas de cada técnica. As estratégias atuais disponíveis para o tratamento dessa doença incluem cirurgia aberta convencional, cirurgia videolaparoscópica e tratamento endovascular^1^^-^ 4. Apesar do entusiasmo inicial com a cirurgia aórtica laparoscópica, essa técnica não obteve ampla aceitação em cirurgia vascular, em decorrência dos desafios inerentes ao procedimento e da extensa curva de aprendizado, ficando seu uso restrito a poucos centros especializados^2^^,^ 3. Existem evidências que sugerem que a cirurgia laparoscópica eletiva para a correção do AAA apresente invasividade comparável à da cirurgia endovascular (EVAR), com as vantagens de menor taxa de conversão, e similar morbimortalidade, além de ser opção minimamente invasiva para o tratamento dos pacientes com anatomia desfavorável para EVAR^1^^,^ 3 ^,^ 4.

Nas cirurgias aberta convencional e laparoscópica, é necessário o posicionamento de pinças hemostáticas (clampes atraumáticos) para interrupção de fluxo e refluxo. Porém, apesar de ditos “atraumáticos”, esses clampes são responsáveis por lesões agudas na parede arterial. O grau de lesão parece ser dependente da pressão aplicada pelo clampe e do tempo de clampeamento, e varia desde distorção da íntima até completo desarranjo da camada média do vaso, com enfraquecimento da parede arterial, hiperplasia intimal e reestenose^5^. Margovsky et al.^6^ observaram formação de cavidades na camada média, sendo essa alteração conhecida como necrose cística da média e encontrada em processos degenerativos da aorta como dissecções aórticas, aneurismas degenerativos e envelhecimento^7^^,^ 8. Loh et al.^9^ descreveram rotura aguda da aorta abdominal provocada pelo clampeamento, documentando lesão parietal isquêmica localizada que provocou enfraquecimento da integridade estrutural da aorta. Por outro lado, observou-se que, apesar de o clampeamento provocar alterações morfológicas^10^, não houve alteração significativa das propriedades mecânicas da parede arterial em longo prazo^11^. Entretanto, faltam estudos que correlacionem as alterações agudas provocadas pelo clampeamento aórtico com as alterações mecânicas da sua parede.

Apesar de não haver clampeamento aórtico durante o tratamento endovascular de rotina, há oclusão temporária do fluxo aórtico pelos dispositivos de liberação das endopróteses e pela insuflação do balão de acomodação após liberação do dispositivo^12^^,^ 13. O efeito negativo do sobredimensionamento da endoprótese sobre as propriedades mecânicas da parede aórtica já foi estudado^14^. Existem cenários clínicos que necessitam insuflação prolongada do balão sem os efeitos do contato da endoprótese sobre a parede arterial – por exemplo na oclusão ressuscitativa por balão Endovascular da aorta (*resuscitative endovascular balloon occlusion of the aorta*, REBOA), utilizada no tratamento das roturas aórticas ou de complicações durante o procedimento^13^^,^ 15. As lesões provocadas pela insuflação de balão intraluminal incluem desde danos ao endotélio (abrasão e dissecção) até necrose da camada média, interferindo mormente na função dos componentes da matriz extracelular^16^. Keris et al.^17^ observaram, em segmentos arteriais submetidos a angioplastia com balão, a redução do módulo elástico tangencial da parede arterial na direção circunferencial, o que poderia predispor ao aumento do diâmetro do vaso quando submetido a condições normais de pressão arterial.

Embora haja aparente benefício nos métodos menos invasivos sobre as repercussões sistêmicas do trauma cirúrgico convencional, são necessários estudos que elucidem as respostas fisiopatológicas do organismo após abordagem cirúrgica aberta, endovascular ou videolaparoscópica da aorta^3^. O objetivo deste estudo foi avaliar comparativamente as alterações estruturais e biomecânicas da parede aórtica provocadas pelos métodos de interrupção temporária de fluxo aórtico, dependendo do acesso cirúrgico utilizado para a abordagem da aorta.

## MATERIAIS E MÉTODOS

Foi feito estudo experimental prospectivo e randomizado. Este estudo seguiu os preceitos do *Guide for care and use of laboratory animals* e foi aprovado pelo Comitê de Ética em Experimentação Animal da Instituição (protocolo 899-2011). Foram usados porcos fêmeas, cruzamento das raças *Large White* e *Landrace*, com peso entre 25 e 30 kg. Após um período de adaptação de 5 a 10 dias, os animais foram aleatoriamente divididos por sorteio simples em três grupos experimentais, com 10 animais cada: Grupo C (cirurgia aberta), Grupo L (videolaparoscopia) e Grupo EV (endovascular). Além desses grupos, foi constituído um grupo suplementar, denominado de Grupo S (*Sham*), composto por 10 animais que tiveram a mesma procedência e a faixa de peso preestabelecida, mas os espécimes de aorta foram retirados pela equipe de estudo logo após o abate no frigorífico de destino do produtor. Esse grupo serviu de padrão de normalidade dos parâmetros biomecânicos e histológicos.

### Procedimentos de anestesia

Os animais foram submetidos a um período de 8 horas de jejum pré-operatório. A medicação pré-anestésica adotada consistiu em uma combinação de 0,1 mg/kg de acepromazina 1%, 8 mg/kg de cetamina, 0,5 mg/kg de xilazina e 0,5 mg/kg de morfina por via intramuscular. Após 15 minutos da medicação pré-anestésica, a veia central da orelha foi canulada para que se fizesse a indução anestésica por administração de 2 mg/kg de cetamina e 2 mg/kg de diazepan. O animal era então colocado em posição pronada na mesa cirúrgica para intubação orotraqueal. A manutenção anestésica foi realizada com isofluorano na taxa de 5-10 mL/kg/min. A ventilação mecânica foi efetuada com volume corrente de 12-15 mL/kg de oxigênio, à razão de 10 a 12 movimentos respiratórios/min, para manter a pressão expiratória de gás carbônico entre 35 e 45 mmHg. A hidratação basal foi realizada com solução de Ringer lactato infundida a 5 mL/kg/h em bomba de infusão intravenosa e completada com a infusão de soro fisiológico 0,9% de acordo com a necessidade hemodinâmica identificada pelo anestesiologista veterinário ao longo do procedimento. A monitorização intraoperatória consistiu em oximetria de pulso por sensor localizado na língua do animal, termômetro transretal para aferição da temperatura corporal, e monitorização invasiva da pressão arterial por meio de acesso carotídeo, onde foi realizado cateterismo arterial com introdutor 6F de 11 cm.

### Procedimentos cirúrgicos

**Grupo C:** Os animais foram posicionados na mesa cirúrgica em decúbito dorsal horizontal. Após antissepsia e delimitação do campo cirúrgico, procedeu-se à laparotomia mediana xifopúbica e à exposição da aorta por via transperitoneal. A exposição da aorta infrarrenal fez-se a partir do cruzamento com a veia renal esquerda e da identificação da origem das artérias renais e da bifurcação aórtica. A seguir, foi posicionada pinça hemostática atraumática de De Bakey na aorta infrarrenal para interrupção do fluxo aórtico pelo período de 60 minutos.

**Grupo L:** Os animais foram posicionados na mesa cirúrgica em decúbito lateral direito. Realizou-se a aplicação da solução antisséptica e delimitação do campo cirúrgico. O pneumoperitônio foi iniciado por meio de punção percutânea com agulha de Veress. Após a realização de pneumoperitônio com CO_2_ com pressão de 16 mmHg, um trocarte de 11 mm foi posicionado lateralmente à cicatriz umbilical para a introdução da lente óptica de 30°. Após o reestabelecimento da pressão de 12 mmHg, dois outros trocartes de 11 mm foram posicionados lateralmente à linha média, acima e abaixo da linha do umbigo. Outros três trocartes de 11 mm foram posicionados ao longo do lado esquerdo da parede abdominal, tendo por referência o rebordo costal, a linha axilar média, o músculo grande dorsal e a crista ilíaca. A exposição da aorta iniciou-se por meio da mobilização medial do cólon esquerdo, do rim esquerdo e da flexura esplênica, utilizando-se de pinças laparoscópicas do tipo *graspers*, tesouras e bisturi harmônico (Ultracision®, Johnson & Johnson®). Após completada a dissecção da aorta abdominal, uma pinça aórtica hemostática laparoscópica (Storz®) foi posicionada imediatamente abaixo da artéria renal esquerda para interrupção do fluxo aórtico pelo periodo de 60 minutos (Figura 1).

**Figura 1:** Local de interrupção de fluxo aórtico nas técnicas minimamente invasivas. (A) Dissecção laparoscópica e identificação da artéria renal esquerda; (B) Posicionamento de pinça hemostattica laparoscópica; (C) Aortografia para identificação da emergência das artérias renais; (D) Aortografia com balão insuflado.

**Grupo EV:** Os animais foram posicionados na mesa cirúrgica em decúbito dorsal horizontal. Após a antissepsia da região inguinal, foi delimitado o campo cirúrgico. Por meio de inguinotomia oblíqua, a artéria femoral comum esquerda foi acessada. Sob visão direta, realizou-se a punção da artéria femoral comum com agulha de corpo único de 21G e passado guia metálico rígido ponta “J”, permitindo cateterismo arterial com introdutor 6F de 11 cm pela técnica de Seldinger. Após a progressão de fio guia hidrofílico 0,035” Roadrunner (Cook Medical®) de 260 cm até a aorta infrarrenal, guiada por radioscopia (Arco Cirúrgico Phillips®, modelo BV 300, EUA), foi introduzido cateter angiográfico de Pig Tail 5F (Cook Medical®) para realização de aortografia, mediante injeção de 20 mL de contraste não iônico Optiray®, com delimitação das artérias renais por *road mapping*. Foram realizadas aortografias pré e pós-insuflação do balão, totalizando 40 mL de contraste. O cateter angiográfico, após retirada do introdutor e hemostasia por compressão manual, foi substituído por cateter-balão complacente de 32 mm de diâmetro para oclusão aórtica (Coda Balloon Catheter®, Cook Medical®, USA). O cateter angiográfico foi então introduzido pelo acesso carotídeo. Após o balão ser posicionado logo abaixo da origem das artérias renais, foi insuflado até interrupção total do fluxo – utilizando-se 15 mL de solução de contraste ao meio –, com controle angiográfico, e mantido pelo período de 60 minutos (Figura 1).

### Protocolo experimental

Todos os procedimentos cirúrgicos foram executados pela mesma equipe e seguindo a mesma sequência experimental. Precedendo-se ao clampeamento aórtico, foi realizada administração endovenosa de heparina sódica na dose de 100 UI/kg em todos os grupos. Em todos os grupos, o período de interrupção de fluxo aórtico adotado foi de 60 minutos. Após esse período, os animais foram eutanaziados por overdose de anestésicos e realizou-se laparotomia mediana para acesso à aorta e coleta de material de estudo. Foram coletados segmentos de aorta abdominal de aproximadamente 5 mm de extensão no sítio de clampeamento/balonamento de cinco animais por grupo, incluindo-se o local onde o clampe/balão foi posicionado, sendo em seguida fixados em formalina tamponada a 10%, para posterior estudo histológico. Os outros cinco animais de cada grupo forneceram amostras de aortas suínas de aproximadamente 3 cm (sendo 1 cm suprarrenal e 2 cm infrarrenal para incluir a área de interrupção de fluxo), as quais foram destinadas ao ensaio de tração.

### Histologia

Os segmentos de aorta coletados e conservados em formol foram processados em Autotécnico Leica TP102 e montados em blocos de parafina em equipamento Leica EG 1160. Posteriormente, foram realizados cortes seriados de aproximadamente 5 µm em micrótomo Leica RM 2155, os quais foram colocados em lâminas de vidro e corados pelas técnicas de hematoxilina e eosina (H&E) e Picrosirius Red, para colágeno, e de Verhoeff, para avaliação das fibras elásticas da parede aórtica. Foram verificadas as alterações gerais da parede vascular como perda da arquitetura lamelar, redução das células musculares lisas, infiltrado celular mononuclear, desorganização das fibras colágenas, intensidade de coloração pelo Picrosirius, e redução ou fragmentação das fibras elásticas.

### Ensaio biomecânico

Os segmentos da aorta abdominal, incluindo as porções imediatamente acima e imediatamente abaixo do local de interrupção de fluxo, foram submetidos a ensaio destrutivo de tração uniaxial de acordo com o método utilizado previamente^8^^,^ 18 ^-^ 20 para avaliação de propriedades mecânicas. As extremidades de cada segmento estudado foram fixadas às presilhas da máquina, placas de metal liso não cortante, permitindo o estiramento longitudinal do segmento aórtico. A velocidade adotada para a aplicação da tração foi de 30 mm/min. O equipamento utilizado foi a Máquina Universal de Ensaios Mecânicos EMIC®, modelo DL 10.000 (Equipments and Testing Systems, Ltd., Curitiba, PR, Brasil), que tem sistema com precisão em torno de 0,018+F/3700 KN, apurada pelas especificações da Associação Brasileira de Normas Técnicas (ABNT) NBR6156 e NBR6674. A máquina opera em conjunto com um microcomputador sob o sistema operacional Windows 98®, utilizando o programa Mtest 1.00. O programa fornece ao final do ensaio valores das propriedades mecânicas escolhidas pelo usuário, além do diagrama de carga *versus* deformação. Esses diagramas possibilitaram a obtenção dos seguintes parâmetros (Figura 2): **Limite de proporcionalidade (N)**: valor máximo de carga em que o material guarda a capacidade de retornar ao seu comprimento original quando interrompido o carregamento; graficamente, corresponde ao valor máximo de tensão de uma função linear na curva carga-deformação, ainda obedecendo à Lei de Hooke, calculado pelo método de Johnson; **Coeficiente de rigidez (N/mm)**: divisão da força (N) pela deformação (mm) correspondentes ao limite elástico; por se tratar de uma relação numérica linear e constante, representa a capacidade de deformação do material conforme a carga aplicada; e **Carga máxima** – **força de rotura (N):** maior carga suportada pelo material até a ruptura, ou seja, o limite de resistência.

**Figura 2:** Modelo matemático utilizado para o ensaio de tração da aorta. (A) Segmento de aorta abrangendo a área de clampeamento, fixada nas presilhas do aparelho; (B) Máquina Universal de Ensaios Mecânicos do Laboratório de Ensaios Biomecânicos do Laboratório de Técnica Cirúrgica e Cirurgia Experimental e o vetor de força uniaxial aplicado na amostra; (C) Diagrama de carga *versus* deformação; (D) Apresentação das fases elástica e plástica no diagrama; (E) Limite de elasticidade calculado pelo método de Johnson; (F) Exemplo de gráfico obtido no ensaio.

### Avaliação estatística

O tamanho amostral de 10 animais por grupo experimental foi calculado com suporte do Escritório de Apoio à Pesquisa da instituição, bem como com base nos estudos experimentais de cirurgia aórtica em modelos suínos^21^^-^ 24, e foi adotado como referência para a composição dos grupos. Primeiramente, foi realizado um teste de normalidade nos dados, e constatou-se que estes apresentavam simetria. Desse modo, realizou uma análise de variância (ANOVA), seguida do teste de Tukey para as comparações múltiplas, a fim de verificar se havia diferença entre os grupos C, L, EV e S.

## RESULTADOS

### Ensaio biomecânico

O grupo EV mostrou-se mais resistente à carga, com maiores coeficiente de rigidez (p < 0,05), carga máxima (p < 0,05) e limite de proporcionalidade (p < 0,05), quando comparado aos grupos C e L. As amostras de aorta do grupo EV tiveram comportamento mecânico similar ao do grupo S no teste de tração (Figuras 3, 4 e 5).

**Figura 3:** Comparação absoluta entre os grupos quanto ao parâmetro carga máxima, em que a diferença de carga máxima foi estatisticamente significativa (p < 0,05) entre os grupos EV e L e entre EV e C.

**Figura 4:** Comparação absoluta entre os grupos quanto ao parâmetro elasticidade, em que a diferença de limite de elasticidade foi estatisticamente significativa (p < 0,05) entre os grupos EV e L e entre EV e C.

**Figura 5:** Comparação absoluta entre os grupos quanto ao parâmetro coeficiente de rigidez, em que a diferença de coeficiente de rigidez foi estatisticamente significativa (p< 0,05) entre os grupos EV e L e EV e C.

### Avaliação histológica da aorta

A histologia da aorta apresentou-se preservada em todos os casos avaliados. Dessa maneira, não foi observada alteração celular, de fibras colágenas ou de fibras elásticas nas amostras avaliadas, independentemente do grupo de estudo (Figura 6).

**Figura 6:** Secções histológicas de aorta abdominal. (A, B, C) Observa-se arranjo lamelar preservado, caracterizado por padrão paralelo das fibras da túnica média. Número e núcleo de células musculares lisas preservados. Ausência de infiltrado inflamatório mononuclear (Hematoxicilina e eosina, 200x); (D, E, F) Fibras colágenas organizadas e coradas normalmente (Picrosirius, 200x); (G, H, I) Fibras elásticas sinuosas presentes em todo o segmento vascular, sem fragmentação e em número preservado (Verhoeff, 200x).

## DISCUSSÃO

Os principais achados desde estudo consistem em alterações provocadas pela pinça hemostática cirúrgica no sítio de interrupção de fluxo, que tem seu uso inevitável durante a cirurgia aórtica. Sempre que um vaso é manipulado, existe possibilidade de rotura de placas, lesão intimal e formação de trombos durante e após o posicionamento de pinças hemostáticas. Mesmo na cirurgia endovascular, 1 hora após balonamento arterial nas angioplastias já ocorrem alterações na parede arterial, tais como: denudação endotelial, deposição de plaquetas, trombos murais e rachaduras endoteliais envolvendo a lâmina elástica interna^25^. O balão utilizado durante EVAR é complacente; porém, permaneceu insuflado acima da pressão arterial do animal por tempo prolongado, e desse modo poderia ser fonte de lesão na parede da artéria. Quando esta é submetida a angioplastia com balão, está sujeita a tensões radiais além do seu intervalo fisiológico; desse modo, poderiam ocorrer danos, em especial nas fibras de colágeno^26^. A complacência do balão fez com que a área de contato do balão com a parede aórtica fosse maior que sua superfície nominal, em razão de sua acomodação ao diâmetro reduzido da aorta dos suínos. Experimentos demonstraram que essa lesão induziria o espessamento da parede do vaso e seria determinada pelo estresse na superfície da parede que tangencia o balão no vaso^27^. Consigny et al.^28^ observaram de imediato aumento do diâmetro arterial, denudação endotelial, lesões em células musculares lisas, diminuição da espessura arterial, e aumento do módulo elástico logo após balonamento arterial. No presente estudo, o segmento aórtico em contato com o balão poderia ser sede de alterações isquêmicas locais, pela compressão dos *vasa vasorum*, por falta de contato da aorta com o sangue circulante e pela reperfusão.

A hipótese de alterações agudas na parede da aorta após balonamento prolongado teria fundamento, mesmo com balão complacente, pois este seria insuflado com pressões altas e por tempo prolongado. Porém, o comportamento mecânico das aortas que tiveram o fluxo interrompido pela insuflação de balão foi similar ao daquelas do controle de normalidade. Esses resultados podem sugerir que a insuflação de balão complacente na aorta, mesmo por tempo prolongado, não provoca alterações parietais estruturais, o que é fundamental para a durabilidade das técnicas endovasculares. O aumento de diâmetro aórtico por eventual lesão e enfraquecimento de parede no sítio de balonamento (fixação proximal das endopróteses) poderia levar à degeneração do colo e a consequentes *endoleaks* tipo I e migração da endoprótese^29^. Ademais, atualmente é cada vez mais difundida a hemostasia endovascular durante emergências aórticas por meio de balão intraluminal pela técnica REBOA^30^, sendo importante obter dados que evidenciem que essa técnica não provocaria alterações mecânicas persistentes da parede aórtica.

As análises mostraram que os parâmetros mecânicos dos grupos C e L foram menores do que os do grupo EV, sendo que as variáveis biomecânicas do grupo S se assemelharam às encontradas no grupo EV, denotando redução da resistência da parede da aorta com o uso de pinças hemostáticas. A distensibilidade da aorta depende dos componentes da túnica média – colágeno, elastina e proteoglicanos – e todos podem afetar a resistência da aorta à tração^8^, de modo que a capacidade elástica dos tecidos cardiovasculares é relacionada diretamente com seu comportamento biomecânico^18^. Testes de resistência à tração refletem, em última análise, a rigidez e a elasticidade da aorta, ou seja, analisam a capacidade do colágeno e da elastina em permitir que a aorta sofra distensibilidade, parte fundamental de sua função^8^. Carga máxima, limite de proporcionalidade e coeficiente de rigidez são os parâmetros mais vinculados a essas propriedades biomecânicas. É possível que esses fatores tenham contribuído para as alterações verificadas nos grupos C e L durante os testes biomecânicos, uma vez que a tensão mecânica da parede arterial depende da carga aplicada e da geometria vascular deformada^31^^-^ 33.

Como os parâmetros biomecânicos da parede arterial são devidos em grande escala ao colágeno e a fibras elásticas, foram incluídas colorações especificas para esses componentes da parede, mas mesmo assim não se observaram alterações marcantes e significativas nesses cortes à microscopia de luz. Borges et al.^7^ demonstraram que a coloração de Picrosirius Red analisada na luz polarizada, somada à microscopia de luz convencional, seria o melhor método para avaliar a estrutura de colágeno, uma vez que permite o estudo do arranjo e da agregação das fibras colágenas, devido à sua birrefringência normal. Essa coloração torna possível a visualização da morfologia de feixes de colágeno íntegro e também de feixes fragmentados, sendo possível também a detecção de colagenólise. Talvez a utilização de luz polarizada pudesse demonstrar algum rearranjo na estrutura tridimensional das fibras colágenas não identificadas nas técnicas convencionais utilizadas.

As análises histológicas não tiveram sensibilidade suficiente para detectar alterações estruturais agudas nos componentes da parede aórtica, mas alterações funcionais ou ultraestruturais poderiam ter ocorrido. Estudos com ultramicroscopia, com histoquímica para outros componentes da média^25^ e com imuno-histoquímica para elastases talvez pudessem mostrar alguma alteração que corroborasse as alterações biomecânicas observadas. Há estudos sugerindo dano ultraestrutural na parede arterial provocado pelo clampe após o ato cirúrgico, mesmo sem lesão histológica identificável à microscopia de luz^34^^,^ 35. As pinças hemostáticas possuem ranhuras, as quais exercem uma pressão pontual significativa sobre a parede arterial, que invariavelmente resulta em trauma nos vasos^24^. A pressão exercida pelo clampe na parede aórtica, além da isquemia transitória local, seguida pela reperfusão, poderia ter provocado alterações na estrutura vascular e nos parâmetros biomecânicos. Embora as alterações agudas da parede aórtica decorrentes do clampeamento sejam documentadas em diversos estudos, aparentemente não resultam em enfraquecimento permanente do vaso^36^. Dobrin et al.^37^ descreveram lesões persistentes da área pinçada por até 6 meses após o ato, mas estas não estavam associadas a alterações mecânicas crônicas da aorta. Esses achados podem explicar a segurança das técnicas que vêm sendo utilizadas há décadas.

## CONCLUSÃO

A utilização de pinça hemostática durante cirurgia aberta ou laparoscópica produziu alterações mecânicas agudas na aorta que sugerem redução de resistência, mesmo sem aparente alteração morfológica. A insuflação prolongada de balão intraluminal não alterou as propriedades mecânicas da parede, denotando manutenção da integridade estrutural.
